# Bionic Nanocoating of Prosthetic Grafts Significantly Reduces Bacterial Growth

**DOI:** 10.1021/acsami.3c18634

**Published:** 2024-03-06

**Authors:** Simon Pecha, Lukas Reuter, Shahabuddin Ohdah, Johannes Petersen, Christiane Pahrmann, Pinar Aytar Çelik, Ahmet Çabuk, Hermann Reichenspurner, Yalin Yildirim

**Affiliations:** † Department of Cardiovascular Surgery, University Heart and Vascular Center, 20246 Hamburg, Germany; ‡ DZHK (German Centre for Cardiovascular Research), Partner Site Hamburg/Kiel/Lübeck, 20246 Hamburg, Germany; § Department of Radiology, University Medical Center Hamburg Eppendorf, 20246 Hamburg, Germany; ∥ Department of Biotechnology and Biosafety, Graduate School of Natural and Applied Science, Eskisehir Osmangazi University, 26480 Eskisehir, Turkey; ⊥ Department of Biology, Faculty of Science and Letter, Eskişehir Osmangazi University, 26040 Eskişehir, Turkey

**Keywords:** vascular prosthesis, superhydrophobic coating, bionic, nanotechnology, prosthetic infection, bioluminescence imaging

## Abstract

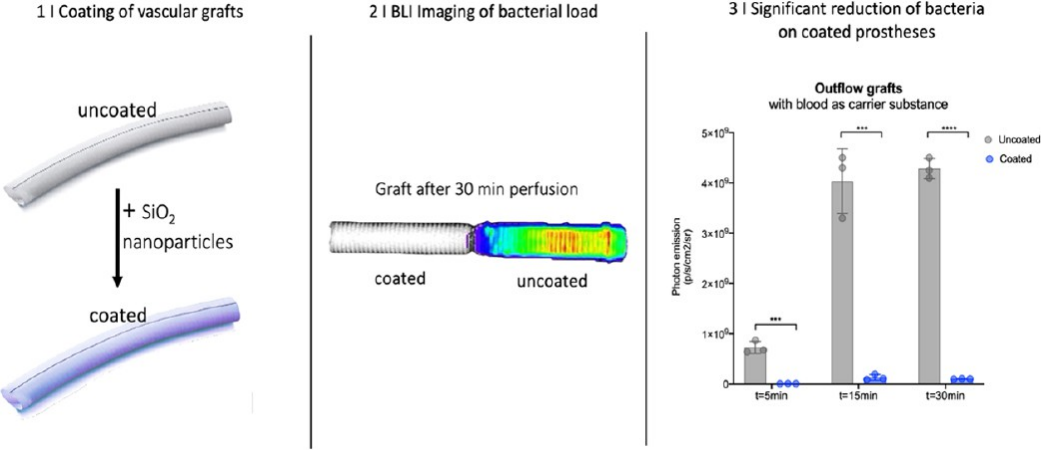

Prosthetic materials are a source of bacterial infections, with significant morbidity and mortality. Utilizing the bionic “Lotus effect,” we generated superhydrophobic vascular prostheses by nanocoating and investigated their resistance to bacterial colonization. Nanoparticles were generated from silicon dioxide (SiO_2_), and coated vascular prostheses developed a nanoscale roughness with superhydrophobic characteristics. Coated grafts and untreated controls were incubated with different bacterial solutions including heparinized blood under mechanical stress and during artificial perfusion and were analyzed. Bioviability- and toxicity analyses of SiO_2_ nanoparticles were performed. Diameters of SiO_2_ nanoparticles ranged between 20 and 180 nm. Coated prostheses showed a water contact angle of > 150° (mean 154 ± 3°) and a mean water roll-off angle of 9° ± 2°. Toxicity and viability experiments demonstrated no toxic effects of SiO_2_ nanoparticles on human induced pluripotent stem cell-derived cardiomyocytes endothelial cells, fibroblasts, and HEK239T cells. After artificial perfusion with a bacterial solution (Luciferase^+^*Escherichia coli*), bioluminescence imaging measurements showed a significant reduction of bacterial colonization of superhydrophobic material-coated prostheses compared to that of untreated controls. At the final measurement (*t* = 60 min), a 97% reduction of bacterial colonization was observed with superhydrophobic material-coated prostheses. Superhydrophobic vascular prostheses tremendously reduced bacterial growth. During artificial perfusion, the protective superhydrophobic effects of the vascular grafts could be confirmed using bioluminescence imaging.

## Introduction

In recent years, the number of medical device implantations has been rising steadily.^1^ In this context, there is also a growing number of device-related infections.^2^ In the United States, more than 1 million device-related infections occur per year, associated with significant morbidity, mortality, and medical costs.^3^ Most commonly, those infections are linked to cardiovascular implants, such as heart valve prostheses,^4^ pacemaker or ICD devices,^5^ left-ventricular assist devices,^6^ or vascular prostheses.^7^ The increasing prevalence of device-related infections leads to widespread use of antibiotics and has resulted in the rapid spread of drug-resistant microbes.^8^ Device infections result from bacterial adhesion at the implantation site with subsequent biofilm formation.^9^ Although very effective in the therapy of bloodstream infections, there is limited potency of antibiotics for biofilm treatment.^10,11^

Several approaches have been used to prevent the bacterial colonization of medical devices. Besides active antibiotic-releasing mechanisms, the use of microbiologicals, such as nitric oxide or silver ions, has been increasingly used.^12,13^ However, these approaches are limited by their potential toxicity, microbiological resistance, and finite release time with restricted efficacy in the long-term run.^14^ Furthermore, physical or chemical modifications of surfaces, such as polymers like polyurethane and poly(ethylene glycol), have been shown to reduce bacterial adhesion in vitro,^15^ but their efficacy in vivo varies with polymer composition, surface chemistry, and bacterial species.

Searching for a different solution, we came across the lotus effect, describing the self-cleaning properties of the lotus flower by its superhydrophobic surface, and tried to incorporate this bionic principle into medical therapy. The “Lotus effect” was first described by Barthlott and Neinhuis in 1997.^16^ This fact is due to its nanoscale rough surface, which decreases the wettability dramatically and thus can also prevent the accumulation of soil for a better photosynthetic performance. Due to the nanoscale characteristics, the surface develops high contact angles because of air entrapment within the rough surface.^17^ These characteristics lead to the surface taking on superhydrophobic properties. A surface is considered hydrophobic from a contact angle of >90° and superhydrophobic from a contact angle of >150°.^18^

By a special coating with silicon dioxide (SiO_2_) nanoparticles, we generated vascular prostheses with structural similarities to this nanoscale roughness to develop resistance against bacterial adhesion. The bacterial colonization of the prostheses was investigated in vitro by high-sensitivity bioluminescence imaging in addition to counting of colony-forming units (CFU) and microscopic analysis. Due to the superhydrophobic nanoscale roughness, the vascular grafts are expected to develop resistance to bacterial colonization and prevent prosthetic infections.

## Methods

### Generation of Nanoparticles

The production of SiO_2_ nanoparticles was carried out by using the modified Stöber method, which is based on an alkoxide sol–gel process. Tetraethylorthosilicate (TEOS) was used as a silica precursor to produce a colloidal sol–gel system, and ammonia was added as a catalyst. A solution of 50 mL of ethanol (Merck, 64175), 10 mL of distilled water, and 3 mL of 28–30% ammonium hydroxide (NH_4_OH; Merck, 1336216) was prepared in a round-bottom flask. 10 mL of TEOS (Evonik, C82604) was added to the solution and stirred at 500 rpm for 1 h at room temperature. The solution was heated with stirring to 60 °C and incubated for an additional 4 h to complete the formation of nanoparticles. The solution was centrifuged at 6000 rpm for 10 min to collect the SiO_2_ nanoparticles. The particles were washed twice with ethanol and twice with distilled water to remove impurities. The particles were dried in an oven at 60 °C for 24 h. Optionally, the particles can be dispersed in a solution for further applications. The amount of ammonium hydroxide can be adjusted depending on the desired particle size. Higher ammonium hydroxide content leads to smaller particles, while lower ammonium hydroxide content leads to larger particles.^19^ For the desired effect, the nanoparticles should have an outcome size between 80 and 100 nm. In order to obtain SiO_2_ particles within this range, we added 3 mL of NH_4_OH. The size of the particles was determined after their production using transmission electron microscopy (TEM).

### Nanotechnological Coating

For the coating, we used common implantable Dacron prostheses and patches (Vascutek Terumo, Scotland). The prostheses used, consisting of poly(ethylene terephthalate) (PET), had a length of 10 cm and a diameter of 1 cm, and the patches had a size of 1 cm × 1 cm.

Both the prostheses and patches were coated with the inorganic compound silicon dioxide nanoparticles, which was obtained by chemical precipitation and left to dry. For this purpose, the prostheses/patches were completely wetted once with the SiO_2_ solution as long as there were no more air bubbles around the tissue and then incubated for 24 h in a 15 mL Falcon (Fisher Scientific, USA) within the SiO_2_ solution at room temperature in the dark.

Subsequently, the SiO_2_ nanoparticles polymerized based on van der Waals forces and developed a nanoscale roughness on the surface.

### Surface Analysis

To assess the surface morphology of the coated prostheses, their nanoscale roughness was examined by electron microscopy after coating. The wettability of the superhydrophobic prostheses was additionally analyzed by water contact and roll-off angle and tilt-drop measurements. Therefore, the contact angle was measured by static sessile drop (SSD) analysis by using goniometry. The used drop volume was 2 μL, and the experiments were performed at room temperature (RT).

### Incubation with Bacteria Solution

2 cm^2^ patches of vascular grafts (*n* = 20) with a water contact angle > 150° were incubated with a bacterial solution under rotation for 2 h. The bacterial solution contained the bacteria *Escherichia coli* and *Staphylococcus aureus*, two pathogens that can typically cause bacterial prosthesis infections. As a control group, vascular grafts (*n* = 20) without coating were incubated in the same manner. After 2 h, the patches (uncoated vs coated) were placed on agar plates using sterile forceps and removed immediately. The agar plates were incubated overnight at 37 °C. After incubation, CFU were analyzed for both samples to determine the number of microbial cells. To study bacterial colonization on the prostheses in detail, bacteria (*E. coli*) were labeled with the reporter enzyme firefly luciferase and analyzed by high-sensitivity live bioluminescence imaging (BLI). For this purpose, an *E. coli* strain was transformed with the plasmid PSF-OXB20-FLUC (Sigma-Aldrich, OGS414), which contained the reporter enzyme under the strong constitutive promoter RecA and antibiotic resistance for kanamycin. The expression of luciferase in the bacteria was tested using BLI. Coated (*n* = 8) and uncoated (*n* = 8) vascular prostheses and vascular patches were incubated with luciferase-positive (Luc+) *E. coli* solution under mechanical stress. d-Luciferin (Biosynth AG, Staad, Switzerland) was added as a substrate for the reporter luciferase (d-luciferin potassium salt dissolved in phosphate-buffered saline pH 7.4; dilution: 1:10) and was added to the bacterial solution again every 20 min. The prostheses were measured at *t* = 0, 5, 30, and 60 min after incubation by BLI. In addition, the vascular patches were rinsed with PBS 60 min after incubation, and the rinsing solution was tested for bacterial load by BLI.

### Mock Circulation

By using a Heartware ventricular assist device (HVAD) pump (Medtronic, Dublin, Ireland), we created a mock circulation. The outflow grafts used in these mock circulation experiments were either nanotechnologically coated or uncoated (control), as described above. For these experiments, human heparinized blood with the above-mentioned bacteria (*Luc^–^E. coli*, *S. aureus*, *Luc*^+^*E. coli*) was used. The analysis of the outflow grafts was performed by CFU counting as well as BLI analysis at different time points.

### Cell Culture

To study the effect of nanoparticles on different cells, we used typical human cell types: HEK293T cells, fibroblasts, human induced pluripotent stem cell (hiPSC)-derived cardiomyocytes (CMs), and endothelial cells (ECs). For the production of hiPSC-CMs and hiPSC-ECs, we used a special protocol that allowed us to produce both hiPSC-CMs and hiPSC-ECs from hiPSC by targeted activation and inhibition of the β-catenin/Wnt pathway. Our human iPSCs were provided and characterized by Gibco (Lot. Nr.: 2365526). The hiPSC were cultivated and maintained on Matrigel (BD356231) 1:40 with Knockout DMEM (Life Technologies, Cat. No.: 10829). The incubator conditions used were 37 °C and 5% CO_2_. The hiPSC were cultured in Essential 8 Flex Basal Media (Life Technologies, Cat. No.: A1516901) supplemented with B-27 minus Insulin (Life Technologies, Cat. No.: A1895601). Media change was performed every 24 h until the cells were 90% confluent. The differentiation protocol was performed as described previously.^20,21^ After differentiation, the cells were then analyzed for their characteristics by FACS analysis and immunohistochemistry.

### Toxicity and Viability Assay

To investigate the toxicity of nanoparticles, we incubated different cell types (hiPSC-cardiomyocytes, hiPSC-endothelial cells, fibroblasts, and HEK293T cells) with nanoparticles and analyzed both cell integrity and viability of different types. For the integrity of cells (DAPI integrity assay), we incubated the cells with 4 μL of 4′,6-diamidino-2-phenylindol (DAPI; Invitrogen D1306) at RT for 10 min and then fixed them with paraformaldehyde (PFA) and processed them by immunohistochemistry. The different cells were then analyzed by confocal microscopy (Zeiss Apotome). ImageJ was used to determine the DAPI mean fluorescence intensity (DAPI MFI) for all cells to investigate the cell integrity. Cells with an intact cell membrane and no cell stress should not take up DAPI into the cell, as long as the cells have not been technically permeabilized. The measured DAPI MFI correlates with the DAPI uptake of the cells, which indicates cell stress. As a negative control, we incubated cells with 15% ethanol (EtOH).

To test the viability of the cells, we transduced the cells with the reporter enzyme luciferase. For hiPSCs, we used a lentiviral system (Amsbio; Cat. No.: LVP434) with the integrated luciferase under the human promoter EF1-alpha with resistance to puromycin as a selection marker. Fibroblasts and HEK293T were transduced by the same lentiviral system. The luciferase activity within the cells was tested by BLI. The Luc^+^ hiPSCs were used for differentiation in Luc^+^ hiPSC-derived cardiomyocytes and Luc^+^ hiPSC-derived endothelial cells. We then incubated the luciferase-positive cells (Luc^+^) with nanoparticles for 24 h and validated the different cell types by bioluminescence imaging. For this purpose, 200.000 Luc^+^ cells were plated out in each case, and the wells were previously coated with nanoparticles and measured. The measured total photon emission correlates with the number of living cells. Thus, the BLI viability assay can be used to obtain information about the number of living cells after incubation with the nanoparticles. Again, incubation with 15% EtOH served as a negative control.

### Bioluminescence Imaging

Bioluminescence imaging (BLI) enables detection of living cells that have previously been labeled with the reporter enzyme luciferase. In this process, the enzyme luciferase converts its substrate luciferin, resulting in photon emission, which can be further analyzed by a BLI measurement.

BLI was used to analyze bacterial colonization on coated and uncoated protheses as well as cell viabilty. Prostheses and rinsing solution were imaged using an IVIS 200 System (PerkinElmer, Waltham, USA). The bioluminescence (photon emission) was detected in units of maximum photons per second per square centimeter per steradian [p/s/cm^2^/sr]. The signals and region of interest (ROI) settings were measured by using Living Image 3.1 software (Media Cybernetics, Rockville, USA).

### Statistical Analysis

Statistical analysis was performed by Graph Pad Prism (Prism 8). Data are presented as mean ± SD. *p*-values < 0.05 are considered significant. Data were tested for significant differences (ns *p* > 0.05, *p < 0.05, **p < 0.01, ***p < 0.001, ****p < 0.0001) using two-tailed Student’s *t* test.

## Results

### Surface Analysis

**Figure 1:**
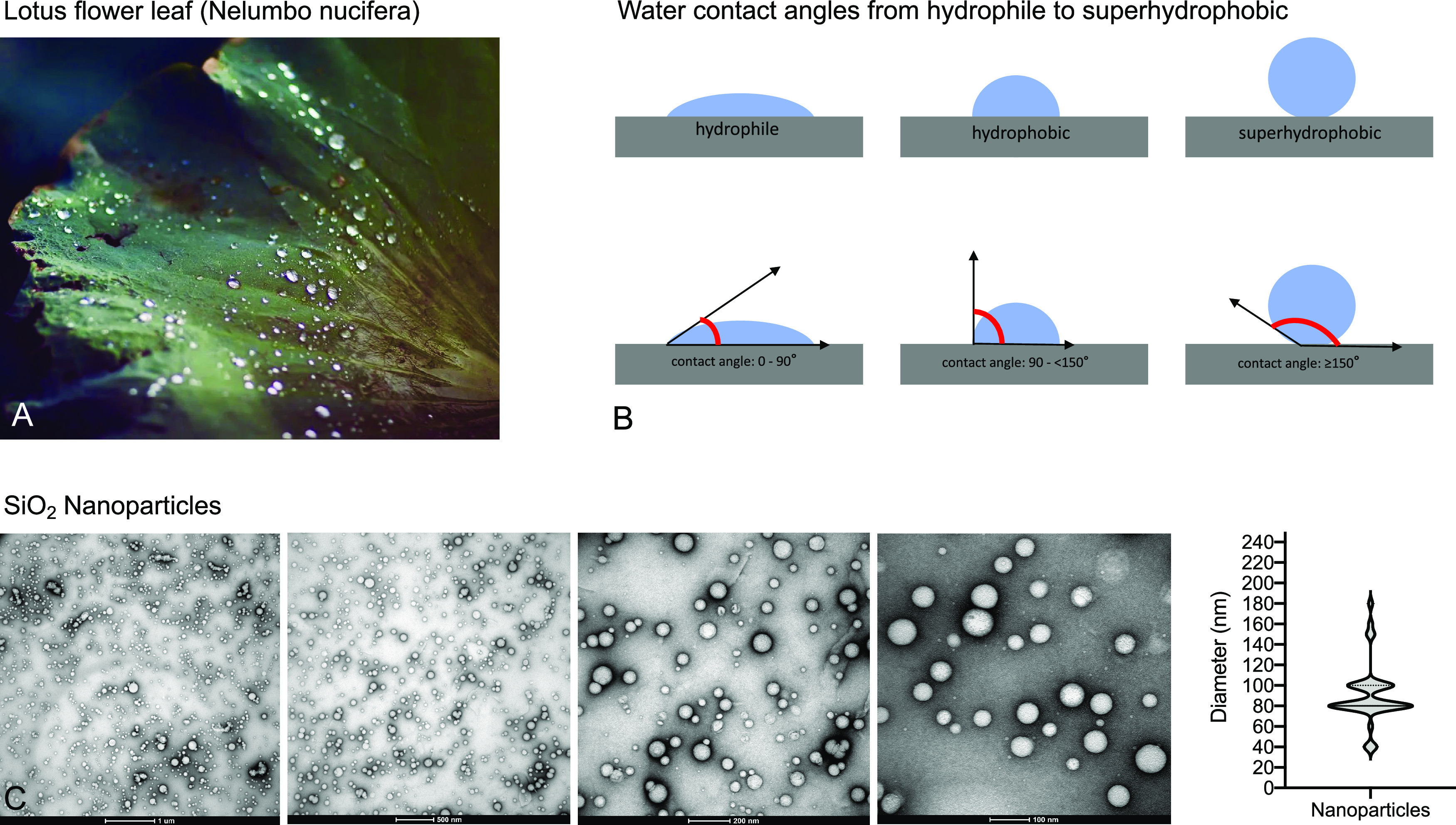
(A) Leaf of the lotus flower with a superhydrophobic surface. (B) Overview of contact angles from hydrophilic to superhydrophobic. (C) Electron microscopy of nanoparticle solution and violin plot of nanoparticle diameters.

**Figure 2:**
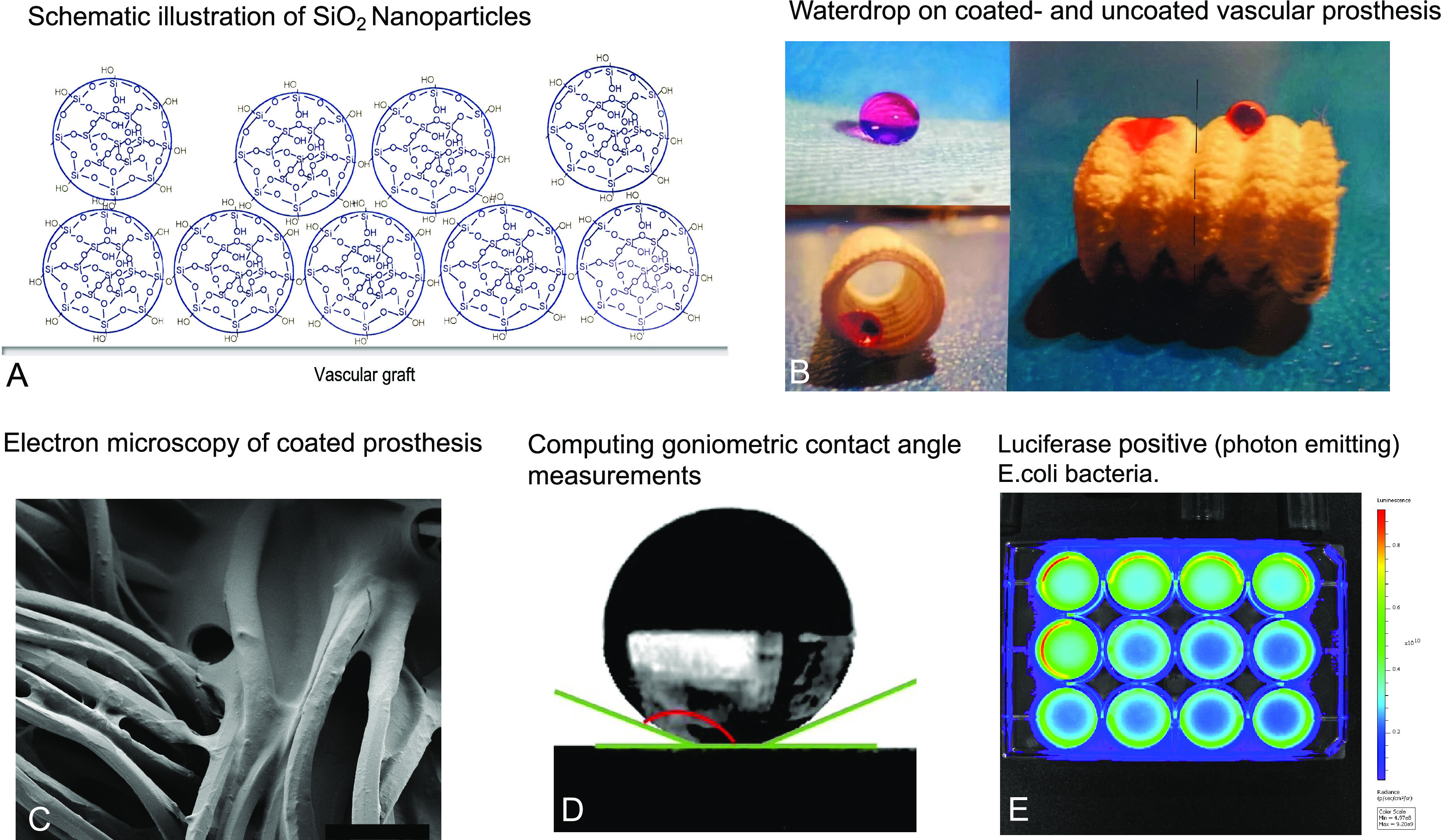
(A) Schematic illustration of nanoparticle formation. (B) Macroscopic properties of a coated vs uncoated prosthesis and behavior of a waterdrop on coated vs uncoated prosthesis. (C) Electron microscopy of a coated prosthesis. (D) Computing goniometric contact angle measurements. (E) Titration series of luciferase-positive (photon emitting) *E. coli* bacteria.

Nanotechnological particles were generated from silicon dioxide (SiO_2_). Isolated electron microscopy analysis of the prepared nanoparticles ranged between 20 and 180 nm (Figure 1). After nanotechnological coating of vascular prostheses, we analyzed every single graft regarding its surface properties. Analysis of contact and roll-off angle by goniometric analysis showed that all coated vascular grafts revealed a water contact angle of > 150° (mean 154 ± 3°) and a mean water roll-off angle of 9 ± 2° (Figure 1). In order to investigate the surface for its nanoroughness, the prostheses were analyzed by electron microscopy. Electron microscopic analysis of the coated prosthetic material confirmed the nanoscale roughness characteristics of superhydrophobic surfaces in comparison to uncoated grafts (Figure 2).

### Bacterial Colonization

**Figure 3:**
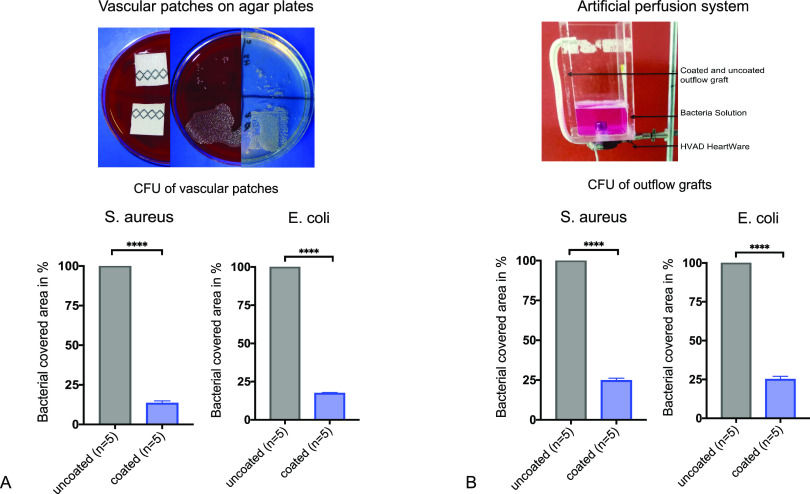
(A) Coated vs uncoated vascular patches on an agar plate and results of colony-forming units counting for *S. aureus* and *E. coli* of coated vs uncoated patches. (B) Artificial perfusion system (Mock circulation) with left-ventricular assist device (LVAD), outflow graft, and LV chamber containing colored bacteria solution. Results of colony-forming units counting for *S. aureus* and *E. coli*. of coated vs uncoated outflow grafts after perfusion with a bacteria solution.

To determine whether the superhydrophobic coating of the prostheses can prevent or minimize bacterial growth, the prostheses were examined for bacterial colonization using CFU and BLI analyses. Investigation of coated grafts with nanoscale roughness proved an 86% reduction of CFU compared to the control group after 24 h incubation in bacteria solution (Figure 2A,B). *S. aureus*-coated patches showed an 87% reduction, and *E. coli*-coated patches showed an 83% reduction of bacterial colonization on the superhydrophobic material. To mimic blood flow in the body, the prostheses (coated vs uncoated) were analyzed by CFU analysis and BLI in a mock circulation using an HVAD. Analysis of our mock circulation experiment by CFU showed 81% reduction of bacterial growth for *S. aureus*-coated outflow grafts and 76% reduction for *E. coli*-coated outflow grafts (Figure 3). For highly sensitive quantification of bacterial colonization, the superhydrophobic prosthetic material was analyzed by live bioluminescence imaging. BLI allows a clear correlation between the current cell number and signal intensity.^22^

Bioluminescence imaging experiments showed a statistically significant reduction of bacterial colonization for superhydrophobic-coated prostheses (Group A) in comparison to untreated controls (Group B) (*t* = 0 min: Group A 5.9 × 10^6^ [p/s/cm^2^/sr] vs Group B 5 × 10^8^ [p/s/cm^2^/sr], *p* < 0.0001; *t* = 5 min: Group A 2.4 × 10^7^ [p/s/cm^2^/sr] vs Group B 7.7 × 10^8^ [p/s/cm^2^/sr], *p* < 0.0001; *t* = 30 min: Group A 3.0 × 10^7^ [p/s/cm^2^/sr] vs Group B 7 × 10^8^ [p/s/cm^2^/sr], *p* < 0.0001; *t* = 60 min: Group A 4.2 × 10^7^ [p/s/cm^2^/sr] vs Group B 7.6 × 10^8^ [p/s/cm^2^/sr], *p* < 0.0001).

Similar results were observed for patches (*t* = 0 min: Group A 9.77 × 10^6^ [p/s/cm2/sr] vs Group B 1.1 × 10^9^ [p/s/cm^2^/sr], *p* < 0.0001; *t* = 30 min: Group A 7.7 × 10^7^ [p/s/cm^2^/sr] vs Group B 1.1 × 10^9^ [p/s/cm^2^/sr], *p* < 0.0001; *t* = 60 min: Group A 6.7 × 10^7^ [p/s/cm^2^/sr] vs Group B 1.7 × 10^9^ [p/s/cm^2^/sr], *p* < 0.0001) after incubation under mechanical stress at all time points (Figure 3A,B).

**Figure 4:**
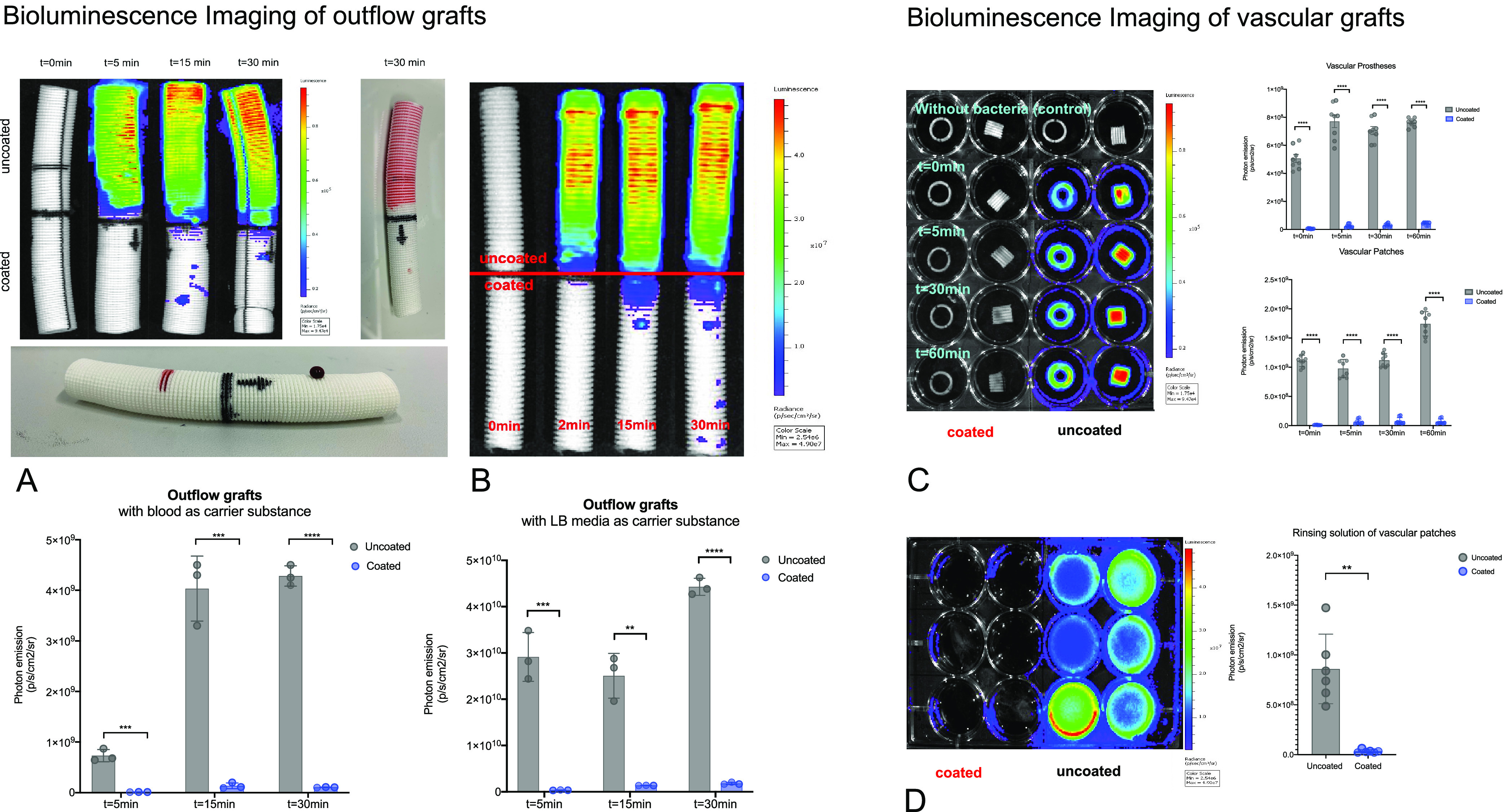
(A) Bacterial colonization with bioluminescence imaging (photon emission) of outflow grafts at different time points after artificial perfusion (mechanical stress) with blood as the carrier substance and analysis of photon emission of coated vs uncoated outflow grafts at different time points. (B) Bacterial colonization with bioluminescence imaging (photon emission) of outflow grafts at different time points after artificial perfusion (mechanical stress) with LB medium as the carrier substance and analysis of photon emission of coated vs uncoated outflow grafts at different time points. (C) Bacterial colonization with bioluminescence imaging (photon emission) of vascular prostheses at different time points after incubation with LB medium as the carrier substance and analysis of photon emission of coated vs uncoated prostheses at different time points. (D) Bioluminescence imaging of rinsing solution after 60 min of coated vs uncoated vascular prostheses and analysis of photon emission of rinsing solution of coated vs uncoated prosthesis.

After the final measurement (*t* = 60 min), the coated vascular prostheses showed a 94% (*p* = 0.0002) and the vascular patches a 96.2% (*p* < 0.0001) reduction in comparison to their uncoated equivalent. BLI analysis of the rinsing solution of both coated and uncoated vascular grafts after 60 min revealed a 96% reduction of bacterial load (*p* = 0.002) (Figure 4).

Our BLI analysis of outflow grafts after mock circulation perfusion with Luc^+^ bacteria in LB media showed a significant reduction of bacterial colonization at all time points (*p*_*t*=2_ = 0.0007; *p*_*t*=15_ = 0.001), a 97% reduction (*p*_*t*=30_ < 0.0001) after the final measurement of mock circulation (Figure 3C). BLI analysis of outflow grafts perfused with Luc^+^ bacteria in heparinized blood showed similar results (*t* = 2 min: Group A 1.1 × 10^7^ [p/s/cm^2^/sr] vs Group B 7.3 × 10^8^ [p/s/cm^2^/sr], *p* = 0.0005; *t* = 15 min: Group A 1.3 × 10^8^ [p/s/cm^2^/sr] vs Group B 4.0 × 10^9^ [p/s/cm^2^/sr], *p* = 0.0005; *t* = 30 min: Group A 1.0 × 10^8^ [p/s/cm^2^/sr] vs Group B 4.2 × 10^9^, *p* < 0.0001) (Figure 4). After bacterial perfusion experiments, additional goniometric analyses were performed to evaluate the superhydrophobic properties after mechanical stress. The end point analysis (after 30 min perfusion) of contact angles showed a reduced water contact angle of 141 ± 6° in comparison to 154 ± 3° before perfusion experiments.

### Toxicity and Viability Assays

**Figure 5:**
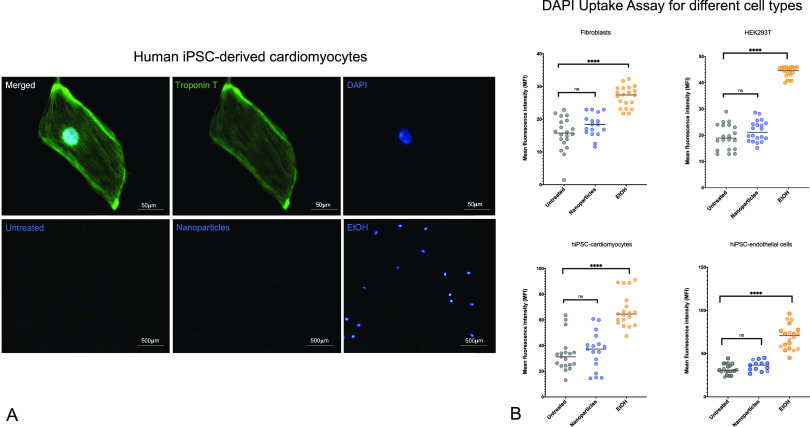
(A) Confocal microscopy of hiPSC-CMs and different conditions. (B) DAPI uptake assay for different human cell types (fibroblasts, HEK293T, hiPSC-derived cardiomyocytes, hiPSC-derived endothelial cells).

To analyze the toxicity of nanoparticles on cells, we investigated the integrity and viability of cells after incubation with nanoparticles. The DAPI integrity assay^23^ showed us that after incubation of cells with nanoparticles for 24 h there was no significant difference in DAPI MFI—which correlates with DAPI uptake and thus the integrity of the cells—between the positive control (no treatment of the cells) and the cells incubated with nanoparticles (*p*_EC_ = 0.0688, *p*_CM_ = 0.6010, *p*_HEK293T_ = 0.1289, *p*_Fibroblasts_ = 0.0822). This was true for all four different cell types; for ECs (MFI_untreated_ = 31.97; MFI_Nanoparticles_ = 35.99; MFI_EtOH_ = 69.38), CMs (MFI_untreated_ = 33.21; MFI_Nanoparticles_ = 35.69; MFI_EtOH_ = 67.54), HEK293T (MFI_untreated_ = 19.43; MFI_Nanoparticles_ = 21.59; MFI_EtOH_ = 44), and fibroblasts (MFI_untreated_ = 15.55; MFI_Nanoparticles_ = 18.51; MFI_EtOH_ = 26.90). The diffence in DAPI MFI between untreated control and negative control with EtOH was significant for all cell types (*p* < 0.0001) (Figure 5).

**Figure 6:**
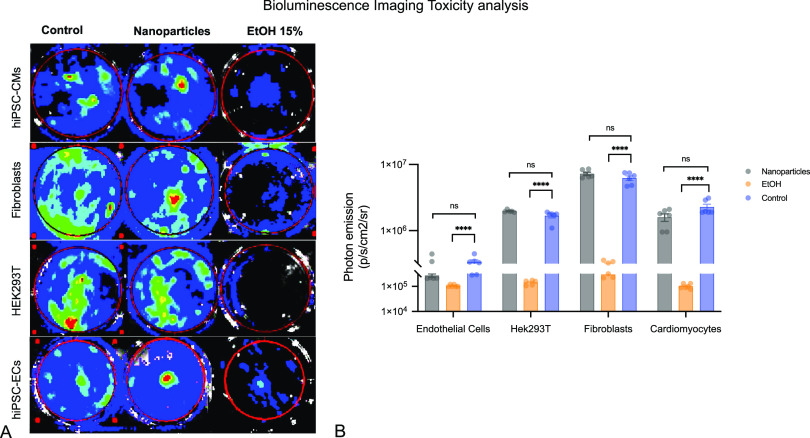
(A) Bioluminescence imaging toxicity assay of different human cell types (fibroblasts, HEK293T, hiPSC-derived cardiomyocytes, hiPSC-derived endothelial cells) with control, nanoparticles, and EtOH. (B) Comparison of photon emission of toxicity assay of different human cell types (fibroblasts, HEK293T, hiPSC-derived cardiomyocytes, hiPSC-derived endothelial cells).

To investigate the survival of cells after incubation with nanoparticles and the toxicity of nanoparticles, these cells were analyzed by BLI. The photon emission signal correlates with the number of living cells.^24^ Cells without metabolism no longer have luciferase enzyme activity due to the ATP-dependent reaction and therefore no longer metabolize the substrate luciferin. For ECs (untreated: 3.37 × 10^5^ [p/s/cm^2^/sr]; nanoparticles: 2.71 × 10^5^ [p/s/cm^2^/sr]; EtOH: 1.05 × 10^5^ [p/s/cm^2^/sr]), for CMs (untreated: 2.27 × 10^6^ [p/s/cm^2^/sr]; nanoparticles: 1.5 × 10^5^ [p/s/cm^2^/sr]; EtOH: 1.02 × 10^5^ [p/s/cm^2^/sr]), for HEK293Ts (untreated: 1.67 × 10^6^ [p/s/cm^2^/sr]; nanoparticles: 1.97 × 10^6^ [p/s/cm^2^/sr]; EtOH: 1.43 × 10^5^ [p/s/cm^2^/sr]), and for fibroblasts (untreated: 6.22 × 10^6^ [p/s/cm^2^/sr]; nanoparticles: 7.24 × 10^6^ [p/s/cm^2^/sr]; EtOH: 2.96 × 10^5^ [p/s/cm^2^/sr]), there was no significance between untreated control and with nanoparticle incubated cells (*p* > 0.05); the difference between control and with EtOH treated cells was significant (*p* < 0.0001) for all four cell types (Figure 6).

## Discussion

Mimicking the bionic “lotus effect,” we successfully generated coated vascular prostheses, showing nanoscale roughness and superhydrophobic properties with a mean water contact angle of 154 ± 3° and a mean water roll-off angle of 9 ± 2°.

Furthermore, for the first time, we were able to demonstrate that a medical prosthetic material with superhydrophobic characteristics significantly reduces bacterial colonization. Both under mechanical stress and during artificial circulation with heparinized human blood, the protective effects of the superhydrophobic coating were confirmed. Bioluminescence imaging experiments of luciferase-positive *E. coli* showed a statistically significant reduction of bacterial colonization for superhydrophobic material-coated vascular grafts in comparison to untreated controls at all time points, with a tremendous reduction in photon emission (bacterial growth) of 97% at the final time point.

One of the most important goals during the implantation of prosthetic material is to avoid bacterial contamination and subsequent infection, a dangerous and potentially lethal complication.^3,25^ There are numerous approaches to structurally modify prosthetic material in such a way that bacterial growth might be prevented or reduced.^12^ Despite many technical advances, bacterial infections of prosthetic material are still considered a major challenge, and in the long term, the biofilm formation on foreign vascular grafts remains a major issue. This can often be inadequately treated only by antibiotic therapy. Patients with infected prostheses must therefore undergo complex reoperations with removal of the infected grafts.^26^

Our data indicate that prostheses with nanoscale roughness and the properties of superhydrophobic surfaces significantly reduce bacterial growth. Since bacteria are present in liquids (water, blood, wound secretions, etc.), it is advantageous that the vascular grafts have reduced contact with these liquids. If the superhydrophobic properties of the surface prevent or at least reduce contact with liquids, the bacteria in the fluid solution cannot reach the surface, and thus, bacterial colonization can be avoided or even at least reduced.

There are two main approaches for developing superhydrophobic surfaces:^27^ developing nanostructures on hydrophobic substrates or chemically modifying a surface, as we did in this study. We coated a vascular prosthesis with SiO_2_ nanoparticles, thereby modifying the prosthesis surface. In this process, van der Waals forces provide active coating of the surface. The coating of the prosthesis with SiO_2_ nanoparticles ensures that the surfaces develop a nanoscale roughness with specific superhydrophobic characteristics. This was demonstrated by surface structural analysis and functional measurements.

To analyze the protective effects of superhydrophobic prosthetic material, coated and uncoated vascular patches were incubated with *E. coli* and *S. aureus*, two pathogens that are responsible for prosthesis infections,^28,29^ for 24 h. The results showed that the superhydrophobic properties of the patches provided significant protection against bacterial growth, as confirmed by CFU analysis. Since small amounts of bacteria were also transferred when the patches were passed from the bacterial solution to the agar plate by sterile forceps, the analysis also showed bacterial colonization of the coated patches. However, the colony-forming units evaluated were not due to the patches but due to the forceps used, which were wetted with bacteria. To solve this technical problem, we used BLI, which allowed us to measure the bacterial colonization of the prostheses without further manipulation. Here, a strong correlation between the number of bacteria and the luminescent signal was seen.

Incubation of the prostheses and patches under mechanical stress showed a highly significant difference in bacterial colonization between coated and uncoated prostheses and patches after 60 min of incubation. The fact that the coating of the grafts withstands mechanical stress for this period is of great importance for the perioperative manipulation of the prostheses as possible bacterial contamination during this time could be prevented by the coating.

Furthermore, we observed that both the coated and uncoated vascular grafts had no significant difference in bacterial colonization between 5 and 30 min (*p* > 0.05) and between 5 and 60 min (*p* > 0.05) of incubation time. This means that within the first 5 min after incubation, the prostheses are completely colonized with bacteria, and no further colonization takes place after this time.

To assess the effect of mechanical stress on coated prostheses after implantation, an artificial circulation was developed using a Heartware left-ventricular assist device to mimic the flow conditions in the human body. It was shown that 30 min of bacterial solution circulation can achieve a 97% reduction in bacterial colonization (both with LB media and with heparinized human blood) of the coated outflow grafts compared to uncoated controls.

However, it was also shown that a noncovalent superhydrophobic coating seems to lose its effect over time.^30^ Thus, in our experiments using the outflow graft prosthesis, 30 min of circulation still provides significant reduction in bacterial colonization and the colonization increases compared to 5 min of perfusion.

Since the superhydrophobic effects used in our study were due to prosthetic coating and not a covalently bonded layer, this fact presents us with further challenges. Ideally, the protective effects would be durable and prevent patients from graft infections lifelong. However, since intraoperative contamination plays an important role in the appearance of peri- and early postoperative wound infections,^31^ the superhydrophobic coating could prevent early graft contamination. Furthermore, the dissolving superhydrophobic effect allows for endothelial in-growth of the vascular grafts, which is also important for the long-term functionality of the prostheses.^32,33^

In the future, other techniques might be investigated to achieve durable superhydrophobic properties of grafts/implanted materials. However, those techniques such as structural surface transformation are technically more demanding and difficult to apply, especially for woven vascular grafts, and also have disadvantages by prohibiting natural reendothelialization of the vascular grafts.

The toxicity of intravenously administered nanoparticles is discussed controversially.^34,35^ However, our in vitro data showed that our nanoparticles had no toxic effects during different toxicity and viability assays in various human cell types. We demonstrated that the cell integrity was not negatively affected by the nanoparticles. The DAPI uptake of nonpermeabilized cells showed no significant differences in comparison to controls. We were able to analyze the cell viability and cell survival by BLI.

BLI analyses showed no significant differences between the controls and cells incubated with nanoparticles. Thus, it can be assumed that no toxic effect on different human-typical cells (cardiomyocytes, fibroblasts, endothelial cells, and HEK cells) could be detected for the nanoparticles.

The used nanotechnological coating is easy to apply and might be suitable for all kinds of foreign materials that are to be implanted in patients, such as pacemaker leads and heart valves but also orthopedic endoprostheses.

## Conclusions

In this study, we have shown for the first time that it is possible to generate superhydrophobic material-coated vascular grafts using SiO_2_ nanoparticles. We furthermore were able to show that the prosthetic material with superhydrophobic characteristics significantly reduces bacterial colonization. Both under mechanical stress of the coated prostheses and during artificial circulation, the shown protective effect of the coating could be confirmed. It is possible that prostheses with superhydrophobic characteristics may help minimize or prevent dangerous and possibly lethal prosthetic infections, especially during intra- and perioperative period.

## Limitation

The limitations of the study are that the experiments are restricted to in vitro analysis.
